# Case Report: Clinical metastasis characteristics of lung adenosquamous carcinoma with *ROS1* rearrangement

**DOI:** 10.3389/fmed.2025.1550130

**Published:** 2025-10-01

**Authors:** Xi Chen, Kewei Ma, Xiaobo Ma, Wenhao Zhu, Bo Liu, Xiumei Duan, Yinghui Xu

**Affiliations:** ^1^Cancer Center, The First Hospital of Jilin University, Changchun, Jilin, China; ^2^Department of Pathology, The First Hospital of Jilin University, Changchun, Jilin, China

**Keywords:** lung adenosquamous carcinoma, *ROS1*, adeno-to-squamous transdifferentiation, tumor immune microenvironment, monoclonal origin

## Abstract

Adenosquamous carcinoma (ASC) of the lung is a rare and aggressive subtype of non-small-cell lung cancer, with a poor prognosis. Previous studies have revealed the existence of numerous oncogenic mutations shared between the adeno and squamous components, thereby implying a potential link between these two pathologies. Nevertheless, the genetic origin and underlying mechanisms of such a connection remain subjects of controversy. Here, we present a remarkable case of ASC where the primary tumor and mediastinal lymph node (LN) metastasis were adenosquamous, while the hilar LN metastasis was pure squamous cell carcinoma. Remarkably, a *ROS1* rearrangement was identified in all lesions, strongly suggesting a common origin for the adeno-squamous components. In other words, ASC represents an intermediate state during the potential transformation from AC to SCC. Through whole-exome sequencing and immunohistochemistry, we analyzed the tumor immune microenvironment and the expression of key lineage-defining transcription factors, including *NKX2-1*, *FOXA2*, and SOX2. Our findings suggest these factors contribute significantly to the adeno-to-squamous pathological transformation. This exceptional case offers valuable insights that could potentially aid in the future recognition and treatment of ASC.

## 1 Introduction

Lung cancer holds the distinction of being the most prevalent cancer worldwide and the leading cause of cancer-related fatalities. Among the various subtypes of non-small cell lung cancer (NSCLC), lung adenosquamous carcinoma (ASC) represents a unique category, accounting for approximately 0.4–4% of cases, exhibiting high malignancy and remarkable plasticity (1–3). The World Health Organization defines ASC as a carcinoma with both squamous cell carcinoma components (SCCC) and adenocarcinoma components (ACC), with each component comprising at least 10% of the tumor under microscopic examination (4). In contrast to lung adenocarcinoma (AC) or squamous cell carcinoma (SCC), ASC, with its mixed adenomatous and squamous pathologies, exhibits elevated recurrence rates and a higher incidence of metastasis. However, the precise underlying mechanism remains elusive, largely due to the complex molecular behavior and unclear origin of clones.

Two major hypotheses have been proposed to explain the histogenesis of ASC: the “collision theory,” which posits those two independent tumors (AC and SCC) merge (5), and the “lineage transition theory,” suggesting transdifferentiation from a single progenitor cell. The advent of next-generation sequencing (NGS) has significantly advanced our understanding of tumor biology. Recent experimental evidence tends to support the notion of pathological transformation occurring within single tumors, based on the observation of identical genetic alterations shared between ACC and SCCC (6–9). Nevertheless, further investigations are warranted to fully comprehend this unusual entity and explore the potential genetic origin and developmental mechanisms underlying this disease.

In this study, we present a unique case of ASC where an identical *ROS1* rearrangement was found in both ACC and SCCC components across primary and metastatic sites, aiming to delve deeper into its genetic origin and underlying mechanisms.

## 2 Case presentation

**Figure 1:**
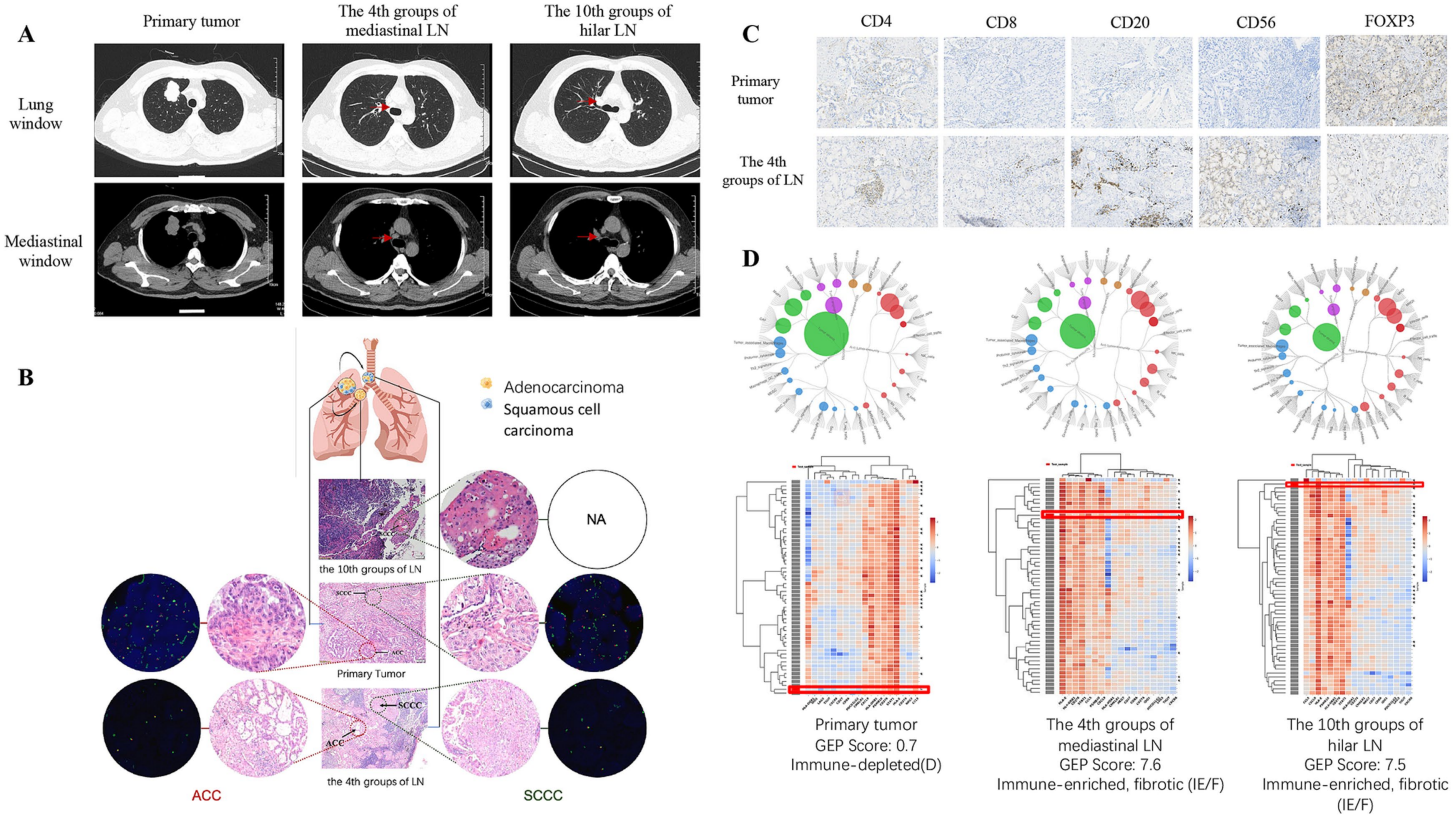
**(A)** Chest CT images showing the primary tumor, the 4th groups of mediastinal LN, and the 10th groups of hilar LN. **(B)** Representative microdissected H&E images from the primary tumor, the 4th groups of mediastinal LN, and the 10th groups of hilar LN, along with confirmation of ROS1 fusion gene by FISH. **(C)** Representative images displaying immunohistochemical staining for CD4, CD8, CD20, CD56, and FOXP3 in the primary tumor, the 4th groups of mediastinal LN, and the 10th groups of hilar LN were not tested due to insufficient samples. **(D)** GEP analysis of the tumor immune microenvironment in each lesion. For each of the three lesions shown, two results are presented. (Top) A circular diagram visualizing the enrichment scores of various immune-related gene sets, which determine the overall GEP score and immune subtype classification. (Bottom) A heatmap visualizing the expression profile of T-cell-inflamed signature genes. In this heatmap, the columns represent the feature genes of the signature. The rows represent different samples; specifically, the row highlighted by the red box indicates the patient’s test sample, which is compared against a reference cohort (other rows) to determine its immune classification. Red indicates higher relative gene expression, while blue indicates lower expression. CT, computed tomography; LN, lymph node; FISH, fluorescence *in situ* hybridization. GEP, gene-expression profiling.

On November 30, 2021, a 50-year-old male with no history of smoking presented at the First Hospital of Jilin University with a persistent cough lasting two months. Chest computed tomography (CT) revealed a 4.8 cm × 3.0 cm mass in the right upper lobe of the lung, along with mediastinal and right hilar lymph node (LN) metastases (Figure 1A). Subsequently, the patient underwent a lobectomy of the right upper lobe on December 3rd, 2021. Post-operative pathological examination confirmed the primary tumor as ASC, measuring 4.5 cm × 3.5 cm × 2.5 cm. Additionally, cancer infiltration was observed in both the 4th group of mediastinal LN and the 10th group of hilar LN. Notably, the proportion of pathological components differed significantly among the three lesions. In the primary lesion, SCCC accounted for 80% and ACC for 20%. In the 4th group of LN, ACC accounted for 80%, and SCCC for 20%. Meanwhile, the 10th group of LN exhibited pure SCCC, with no presence of ACC (Figure 1B). The postoperative pathological stage was pT2bN2M0, IIIA.

Microdissection was performed in regions where ACCs and SCCCs were distinctly separated. Using the amplification refractory mutation system (ARMS) with the AmoyDx Mutations Detection Kit, common driver genes, including *EGFR*, *ALK*, *ROS1*, *KRAS*, *BRAF*, *RET*, *MET*, *HER2*, *NRAS*, and *PI3KCA*, were examined for each lesion. Interestingly, the different lesions shared gene similarities. The primary tumor’s ACC and SCCC, along with the 4th group of mediastinal LN and the 10th group of hilar LN, all exhibited the presence of the *ROS1* rearrangement, while the results of other genetic testing were negative. To validate the presence of the *ROS1* fusion gene, fluorescence *in situ* hybridization (FISH) was performed on sections from ASC samples obtained from the primary tumor and the 4th group of mediastinal LN (Figure 1B). The results also confirmed the presence of the *ROS1* fusion gene, with mutation abundances of 63 and 45%, respectively (see Table 1).

**Table 1:** Gene mutation detection results of the primary tumor, the 4th groups of mediastinal LN and the 10th groups of hilar LN by PCR, FISH and WES.

Lesion	ROS1 by PCR	ROS1 by FISH	ROS1 by WES
Primary tumor	SCCC: (+) ACC: (+)	(+) Abundance: 63%	(+) Abundance: 64.17%
The 4th group of LN	SCCC: (+) ACC: (+)	(+) Abundance: 45%	(+) Abundance: 22.20%
The 10th group of LN	SCCC: (+)	(+)	(+)

LN, lymph node; PCR, polymerase chain reaction; FISH, fluorescence *in situ* hybridization; WES, whole-exome sequencing.

To investigate the impact of the tumor immune microenvironment (TIME) on adeno-to-squamous transdifferentiation (AST), the samples were subjected to immunohistochemical analysis. Tissue sections were stained for CD4, CD8, CD20, CD56, and FOXP3, as depicted in Figure 1C. Upon comparing the changes in the immune microenvironment between the primary focus and metastatic LNs, it was observed that CD4^+^, CD8^+^, CD56^+^, and CD20^+^ were lower in the primary foci, while FOXP3 was higher. To further elucidate the mechanism underlying the AST, whole-exome sequencing (WES) was conducted on each lesion. The analysis revealed the presence of the EZR: *exon10-ROS1: exon34* fusion in all lesions, with no additional mutations detected. Further analysis of the WES data provided deeper insights into the tumor’s biological characteristics. Notably, the expression levels of four key lineage-defining transcription factors, *NKX2-1*, *FOXA2*, *SOX2*, and *TP63*, showed significant differential expression across the three lesions, as summarized in Table 2. Using gene-expression profiling (GEP) to assess the T-cell inflamed signature, the analysis suggested that the primary focus was an “immune desert” type (score: 0.7), while the LNs were an “immune-enriched, fibrotic” type (scores: 7.5–7.6), potentially indicating higher immunoreactivity within the lymphoid tissue (Figure 1D). Gene set enrichment analysis (GSEA) was employed for Hallmark pathway analysis (Supplementary Figure 1). Compared to primary lesion, the expression of genes in the “KRAS_SIGNALING_DN” pathway was significantly downregulated in metastatic lesions.

**Table 2:** Summary of whole-exome sequencing results and TPM levels of four crucial transcription factors involved in pathological transformation.

Lesion	Primary tumor	The 4th group of LN	The 10th group of LN
TMB	0.72 Muts/Mb, TMB-L	0.0 Muts/Mb, TMB-L	0.0 Muts/Mb, TMB-L
NKX2-1	1273.78	155.23	106.58
FOXA2	125.39	31.73	54.94
SOX2	65.76	230.29	945.52
TP63	260.68	80.45	26.92

TPM, transcripts per million.

The identification of a shared, rare *ROS1* fusion gene across both the adenocarcinoma and squamous cell components offers a valuable opportunity to investigate the monoclonal origin of ASC and explore the potential mechanisms underlying its development and intra-tumoral heterogeneity.

## 3 Discussion

In our manuscript, we presented a rare case of ASC where the primary focus and mediastinal LN metastases exhibited adenosquamous components, while the hilar LN metastases were pure squamous. Remarkably, both the ACC and SCCC components in different foci harbored the *ROS1* fusion gene. The role of the characteristic *ROS1* fusion gene as a marker in the development and progression of ASC, as well as the reasons for the change in the ACC to SCCC ratio during metastasis, warrant further in-depth discussion.

### 3.1 The origins and development of ASC

**Figure 2:**
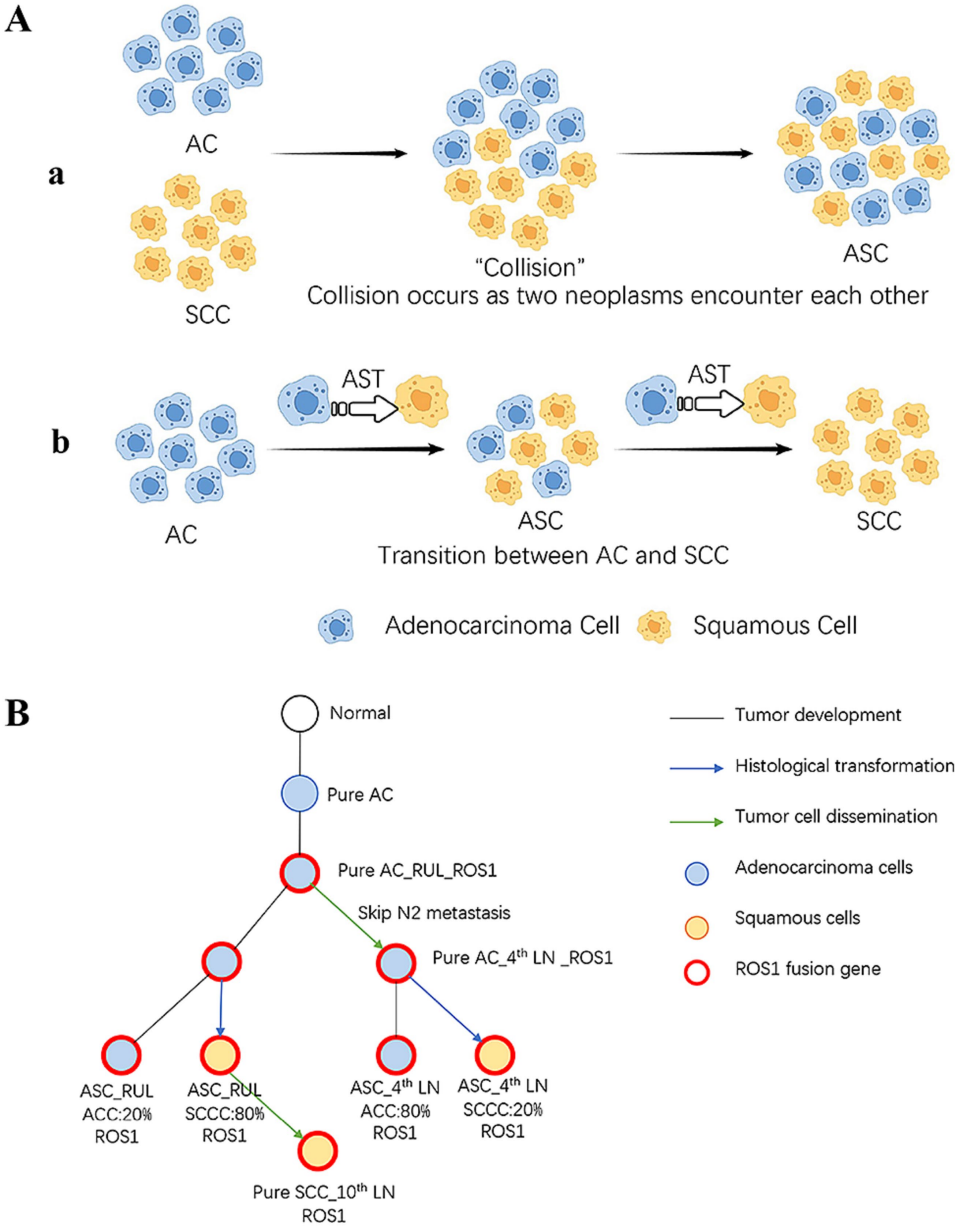
**(A)** Schematic representation of the main theories hypothesizing the origin of ASC. **(a)** The theory of “collision” ASC metastasis and transdifferentiation between AC and SCC. **(b)** The theory of transition between AC and SCC. **(B)** Example phylogenetic trees depicting ASC metastasis and transdifferentiation. ASC, adenosquamous carcinoma; AC, adenocarcinoma; SCC, squamous cell carcinoma; RUL, right upper lobe; SCCC, squamous cell carcinoma components; ACC, adenocarcinoma components; LN, lymph node.

The origin of ASC, presenting mixed glandular and squamous phenotypes, however, is still enigmatic. Two principal theories have been proposed: the “collision theory,” which posits that ASC results from the merging of two separate, independently arising tumors (an AC and an SCC) (10), and the “monoclonal origin theory,” which suggests that both components arise from a single progenitor cell via lineage transition or divergent differentiation (11). Figure 2A briefly depicts the two hypotheses for the origin of ASC. Our case provides a unique opportunity for an explicit comparative analysis of these two hypotheses.

The identification of shared driver mutations is crucial for determining tumor clonality. While previous studies have demonstrated the presence of the identical oncogenic drivers, such as *EGFR* and *KRAS*, in both components of ASC the interpretation can be ambiguous. Because these mutations are relatively frequent in NSCLC, their shared presence could plausibly result from coincidence—two independent tumors arising with the same common mutation—thereby not definitively excluding the collision theory.

The evidentiary weight shifts substantially in favor of the monoclonal theory when identical rare and highly specific genetic alterations are identified in both the AC and SCC components of an ASC. Our study detected the identical *ROS1* fusion gene in both components of the tumors. The *ROS1* fusion gene is a rare genomic alteration detected in only 1–2% of lung adenocarcinomas and extremely rare in squamous cell histology (12–15). The statistical unlikelihood of two separate progenitor cells independently developing the exact same rare *ROS1* fusion and then colliding spatially makes the monoclonal hypothesis, where a single *ROS1*-rearranged progenitor cell undergoes divergent differentiation or transdifferentiation, a far more parsimonious explanation. This type of finding, leveraging a rare mutational event as a clonal marker, offers a more definitive basis for inferring a common cellular origin.

### 3.2 Changes in the proportion of ACC and SCCC

In this case, the proportion of ACC and SCCC components varied among the different lesions. Indeed, metaplastic changes and phenotypic interconversion are commonly observed among various subtypes of lung cancer. It is worth mentioning that most of such transformation were induced by drug treatment resulting in acquired resistance. Interestingly, our patients showed this transformation before receiving any anti-neoplastic treatment, leading to a better understanding of primary AST mechanism.

The varying pathological compositions between the primary tumor and its metastases raise the question of whether the metastatic process itself influences this transformation. LN metastasis in NSCLC typically follows a pathway from local LN in the lung to hilar LN and then to mediastinal LN. Different squamous cell proportions lead us to think transfer pathway in this case is distinct from conventional metastasis. Previous studies have reported approximately 17.2 to 42.7% of N2-NSCLC patients undergoing surgical resection develop skip N2 metastasis, showing mediastinal LN metastases without corresponding peribronchial or ipsilateral hilar LN involvement (16, 17). Additionally, AC appears to be more prone to mediastinal LN skip metastasis than SCC, particularly acinar predominant AC in the right upper lung, which aligns with our current case.

Adeno-to-squamous transdifferentiation can be affected by transcription factor. Previous studies have demonstrated that four lineage-defining TFs, namely NKX2-1, FOXA2, SOX2, and TP63, form a counteracting regulatory network controlling the development of ASC (11). Specifically, NKX2-1 and FOXA2 primarily promote the maintenance of the adenocarcinoma lineage, while SOX2 and TP63 strongly drive squamous lineage differentiation. The dynamic balance of this regulatory network is figuratively described as a “seesaw”; its imbalance leads the cell lineage to tilt to one side. Within this core regulatory network, complex interactions exist among TFs. For example, SOX2 can inhibit NKX2-1 activity, and the loss or downregulation of NKX2-1 (common in SOX2-driven squamous differentiation) further accelerates SOX2-driven SCC development, indicating that NKX2-1 normally has an inhibitory effect on SOX2-mediated AST (18).

The TF expression profile observed in this case study also reflects this complexity: in the hilar lymph node group 10, which presented as pure squamous carcinoma, the transcriptional activity of adeno-lineage TFs FOXA2 and NKX2-1 was significantly weakened, while the squamous-lineage TF SOX2 showed high expression. This is highly consistent with the “seesaw” theory and the antagonistic regulatory network model. The high transcriptional activity of NKX2-1 in the primary lesion, while its expression is low in the pure squamous hilar lymph node, appears contradictory. However, NKX2-1 may play a complex, even paradoxical, role in lung cancer. Some studies indicate that NKX2-1 has tumor-suppressive functions in certain contexts; its loss is associated with metastatic progression in some models, and it can inhibit SOX2-driven squamous carcinogenesis and the AST process (19). Therefore, the relatively high NKX2-1 level in the primary lesion might reflect an ongoing but incomplete transdifferentiation process. Concurrently, the “immune desert” phenotype of the primary lesion (GEP score 0.7) might also be associated with high NKX2-1 expression, as NKX2-1 downregulation is linked to increased neutrophil infiltration (20).

Beyond transcription factors, the tumor immune microenvironment (TIME) is another critical factor influencing AST. The presence of heterogeneity in tumor pathology is a prominent characteristic of tumors, existing between patients, within tumors, and among different tumors (21). A recent study involving 30 ASC patients revealed the existence of TIME heterogeneity between ACCs and SCCCs, which could be linked to branch evolution and selection. Based on these findings, the researchers hypothesized that in SCCCs, high expression of programmed cell death-ligand 1 (PD-L1) can induce immune escape by reduced inflammatory infiltration into tumor cells in this component.

In our case, the TIME of the primary lesion was classified as an immune-depleted (“cold”) subtype, whereas the metastatic LNs were categorized as immune-enriched/fibrotic (“hot”) subtypes. This was further substantiated by immunohistochemical analysis, which showed lower infiltration of CD4^+^, CD8^+^, CD56^+^, and CD20^+^ cells and higher levels of FOXP3^+^ cells in the primary tumor compared to the metastatic LNs. While these IHC findings are illustrative, we acknowledge the inherent challenge in interpreting immune cell staining within the context of a lymph node, where differentiating tumor-infiltrating lymphocytes from the resident lymphoid tissue is difficult. Additionally, GSEA analyses revealed that KRAS signaling was downregulated in the metastatic LNs, implying higher KRAS activity in the primary tumor. This is significant, as KRAS mutations can foster an immunosuppressive TIME by upregulating PD-L1 level and downregulating infiltration of CD8^+^ TILs (22), providing further evidence for the suppressed immune status of the primary lesion.

These findings strongly suggest that the TIME plays a pivotal role in the AST process. However, we must acknowledge that lymph nodes are inherently immune-rich tissues. Therefore, the observed ‘immune-enriched’ phenotype in the metastatic lymph nodes could be partly influenced by the baseline lymphoid stroma, which introduces a potential bias to this interpretation and warrants caution. We hypothesize that the transdifferentiation from AC to SCC is favored in an immunosuppressive microenvironment. In such a “cold” environment, squamous cells may possess a survival advantage over adenocarcinoma cells, ultimately out-competing them and completing the phenotypic switch.

Consequently, we infer that the ASC represents an intermediate state during potential transformation from AC to SCC. The varying SCC proportion of ASC reflects the degree of squamous cell transition. Throughout the tumor growth and development, both AC or SCC cells have the capacity to disseminate and give rise to metastases of either phenotype. During the early stages of our case, the primary lesion was mainly composed of ACC while the *ROS1* fusion gene occurred as a trunk mutation, likely underwent skip metastasis to the mediastinal LNs. As the tumor progressed, intratumoral lineage transition occurred simultaneously, resulting in an increase in the SCC components, with certain characteristics of SCC becoming evident. Metastases of SCC phenotype occurring at advanced stages. Both the primary and metastatic foci underwent AST, whereas the surrounding TIME and other factors contribute to the extent of squamous cell transition, manifested by different SCC proportions. The metastasis and transdifferentiation process is depicted in Figure 2B.

Nevertheless, our study has certain limitations. Firstly, we only had one case, and individual variability may exist. Secondly, compared to the broad sequencing approaches of whole-genome sequencing, we performed WES on the patient, covering less of the genome, which may limit mutational signature analysis on this data. Additionally, the lack of paraneoplastic tissue control in this case may have limitations in the analysis of the microenvironment. Thirdly, the absence of extra-nodal metastases for comparison is a notable limitation of our current study, and future research is needed to validate our hypothesis in non-lymphoid metastatic sites.

## 4 Conclusion

This rare case of ASC with the *ROS1* fusion gene sheds light on the genetic homogeneity of ACC and SCCC, despite the spatial and temporal separation of the primary and metastatic foci. These findings strongly suggest that ASC represents an intermediate state during the potential transformation from AC to SCC, and AC or SCC cells have the capacity to disseminate and give rise to metastases of either phenotype. The TIME is considered to play a prominent role in the AST process. The insights gained from this case may prove valuable in further understanding the genomic origin and unique biological behavior, including the mechanism of transdifferentiation, in ASC. Moreover, this case may have implications for enhancing clinical diagnosis and treatment strategies for ASC in the future.

## Data Availability

The original contributions presented in the study are included in the article/Supplementary material, further inquiries can be directed to the corresponding authors.
