# Supplementary material for: Case Report: Clinical metastasis characteristics of lung adenosquamous carcinoma with ROS1 rearrangement
Source: Front Med (Lausanne). 2025 Oct 1;12:1550130. doi: 10.3389/fmed.2025.1550130 (PMC12521446; doi:10.3389/fmed.2025.1550130)
Supplement: Supplementary file 1 [file Data_Sheet_1.pdf]

Supplementary Material

1.1 Supplementary Figures

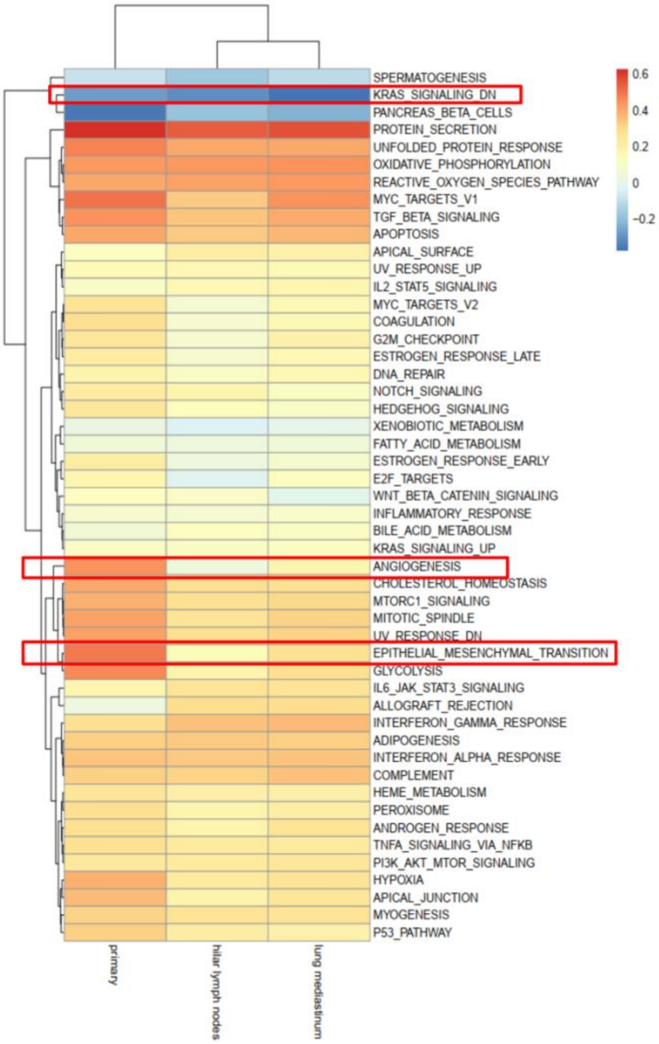

Supplementary Figure 1. Hallmark pathway analysis.

A

	GS follow link to MSigDB	NOM p-val
1	HALLMARK_ANGIOGENESIS	0.049
2	HALLMARK_KRAS_SIGNALING_DN	0.003
3	HALLMARK_ESTROGEN_RESPONSE_LATE	0.014
4	HALLMARK_HEDGEHOG_SIGNALING	0.287
5	HALLMARK_BILE_ACID_METABOLISM	0.285
6	HALLMARK_KRAS_SIGNALING_UP	0.005
7	HALLMARK_ESTROGEN_RESPONSE_EARLY	0.101
8	HALLMARK_EPITHELIAL_MESENCHYMAL_TRANSITION	0.038
9	HALLMARK_FATTY_ACID_METABOLISM	0.277
10	HALLMARK_APICAL_SURFACE	0.472
11	HALLMARK_ALLOGRAFT_REJECTION	0.028
12	HALLMARK_APICAL_JUNCTION	0.240
13	HALLMARK_SPERMATOGENESIS	0.504
14	HALLMARK_COAGULATION	0.327
15	HALLMARK_WNT_BETA_CATENIN_SIGNALING	0.456
16	HALLMARK_INFLAMMATORY_RESPONSE	0.208
17	HALLMARK_TGF_BETA_SIGNALING	0.581
18	HALLMARK_NOTCH_SIGNALING	0.570
19	HALLMARK_ANDROGEN_RESPONSE	0.560
20	HALLMARK_GLYCOLYSIS	0.512

B

	GS follow link to MSigDB	NOM p-val
1	HALLMARK_ALLOGRAFT_REJECTION	0.000
2	HALLMARK_ANDROGEN_RESPONSE	0.160
3	HALLMARK_ANGIOGENESIS	0.149
4	HALLMARK_BILE_ACID_METABOLISM	0.235
5	HALLMARK_KRAS_SIGNALING_DN	0.050
6	HALLMARK_KRAS_SIGNALING_UP	0.009
7	HALLMARK_FATTY_ACID_METABOLISM	0.211
8	HALLMARK_ESTROGEN_RESPONSE_EARLY	0.114
9	HALLMARK_COAGULATION	0.116
10	HALLMARK_ESTROGEN_RESPONSE_LATE	0.112
11	HALLMARK_APICAL_SURFACE	0.545
12	HALLMARK_INFLAMMATORY_RESPONSE	0.063
13	HALLMARK_XENOBIOTIC_METABOLISM	0.276
14	HALLMARK_COMPLEMENT	0.199
15	HALLMARK_EPITHELIAL_MESENCHYMAL_TRANSITION	0.178
16	HALLMARK_IL2_STAT5_SIGNALING	0.223
17	HALLMARK_APICAL_JUNCTION	0.335
18	HALLMARK_NOTCH_SIGNALING	0.576
19	HALLMARK_TNFA_SIGNALING_VIA_NFKB	0.276
20	HALLMARK_HYPOXIA	0.402
21	HALLMARK_WNT_BETA_CATENIN_SIGNALING	0.553
22	HALLMARK_PI3K_AKT_MTOR_SIGNALING	0.475

C

	GS follow link to MSigDB	NOM p-val
1	HALLMARK_WNT_BETA_CATENIN_SIGNALING	0.028
2	HALLMARK_NOTCH_SIGNALING	0.079
3	HALLMARK_KRAS_SIGNALING_DN	0.022
4	HALLMARK_REACTIVE_OXYGEN_SPECIES_PATHWAY	0.234
5	HALLMARK_INFLAMMATORY_RESPONSE	0.001
6	HALLMARK_COAGULATION	0.115
7	HALLMARK_APICAL_SURFACE	0.447
8	HALLMARK_FATTY_ACID_METABOLISM	0.405
9	HALLMARK_XENOBIOTIC_METABOLISM	0.285
10	HALLMARK_HEDGEHOG_SIGNALING	0.530
11	HALLMARK_KRAS_SIGNALING_UP	0.187
12	HALLMARK_SPERMATOGENESIS	0.522
13	HALLMARK_APICAL_JUNCTION	0.334
14	HALLMARK_EPITHELIAL_MESENCHYMAL_TRANSITION	0.347
15	HALLMARK_TGF_BETA_SIGNALING	0.660
16	HALLMARK_ANDROGEN_RESPONSE	0.718
17	HALLMARK_ESTROGEN_RESPONSE_LATE	0.642
18	HALLMARK_HEME_METABOLISM	0.712
19	HALLMARK_G2M_CHECKPOINT	0.727
20	HALLMARK_PI3K_AKT_MTOR_SIGNALING	0.752

Supplementary Figure 1. Gene Set Enrichment Analysis (GSEA) in the HALLMARK datasets. **A**, GSEA for primary tumor and the 10th groups of hilar LN; **B**, GSEA for primary tumor and the 4th

groups of mediastinal LN; **C**, GSEA for the 4th groups of mediastinal LN and the 10th groups of hilar LN.
