# Supplementary figures and images for: Case Report: Clinical metastasis characteristics of lung adenosquamous carcinoma with ROS1 rearrangement
Source: Front Med (Lausanne). 2025 Oct 1;12:1550130. doi: 10.3389/fmed.2025.1550130 (PMC12521446; doi:10.3389/fmed.2025.1550130)

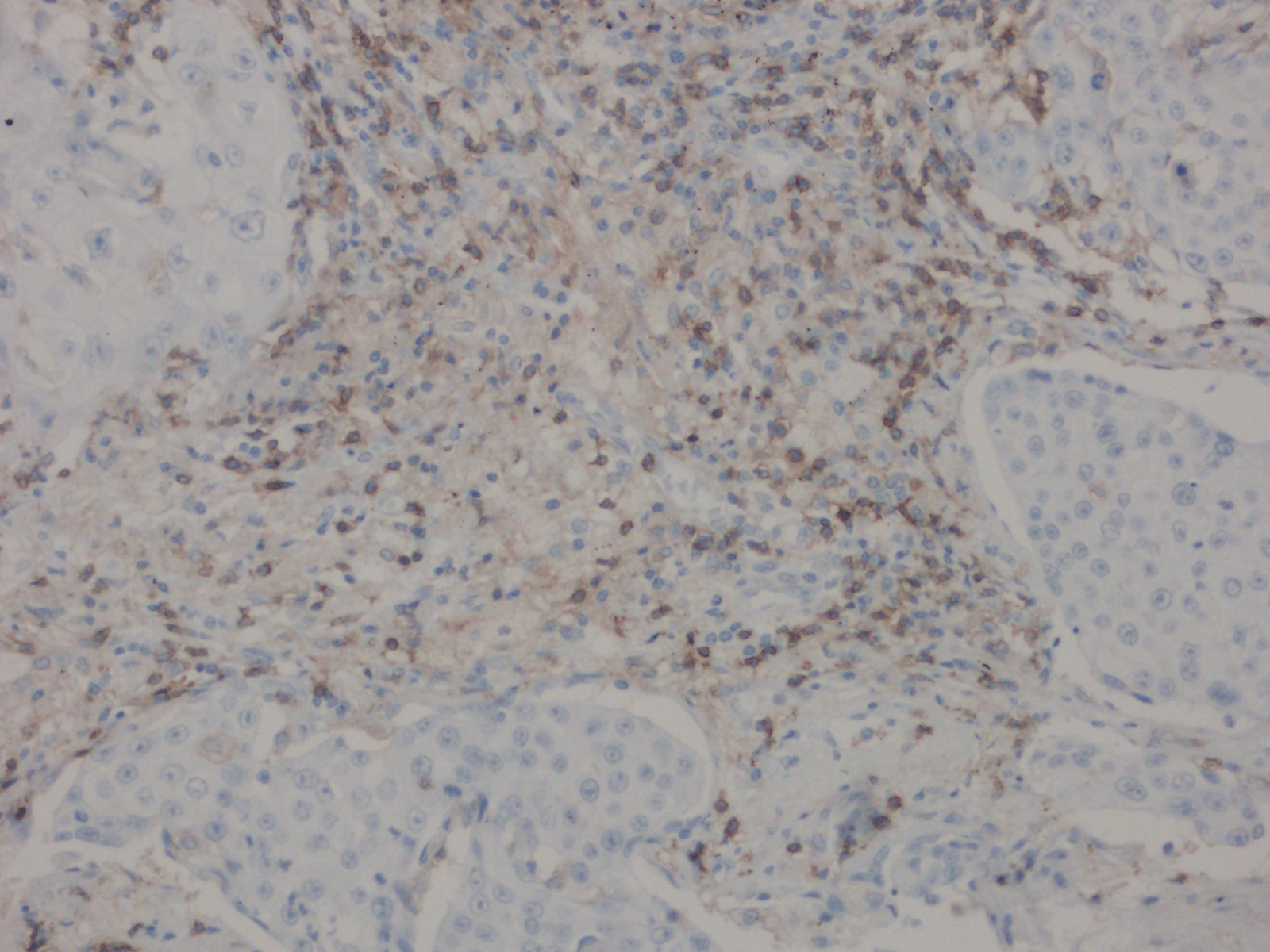

Supplement: Supplementary file 2 [file Data_Sheet_2.zip › primary tumor CD4.tif]

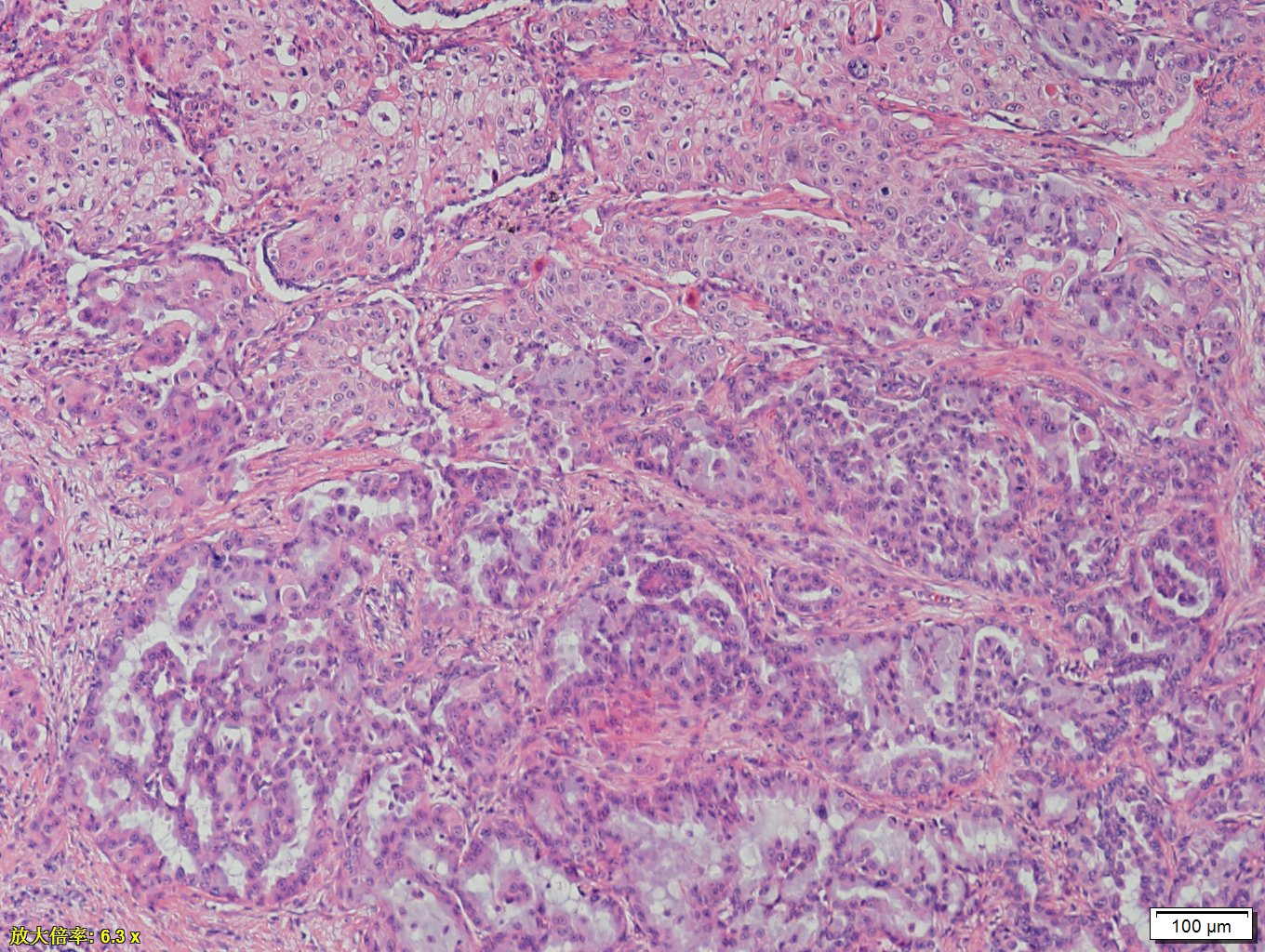

Supplement: Supplementary file 2 [file Data_Sheet_2.zip › primary tumor.jpg]

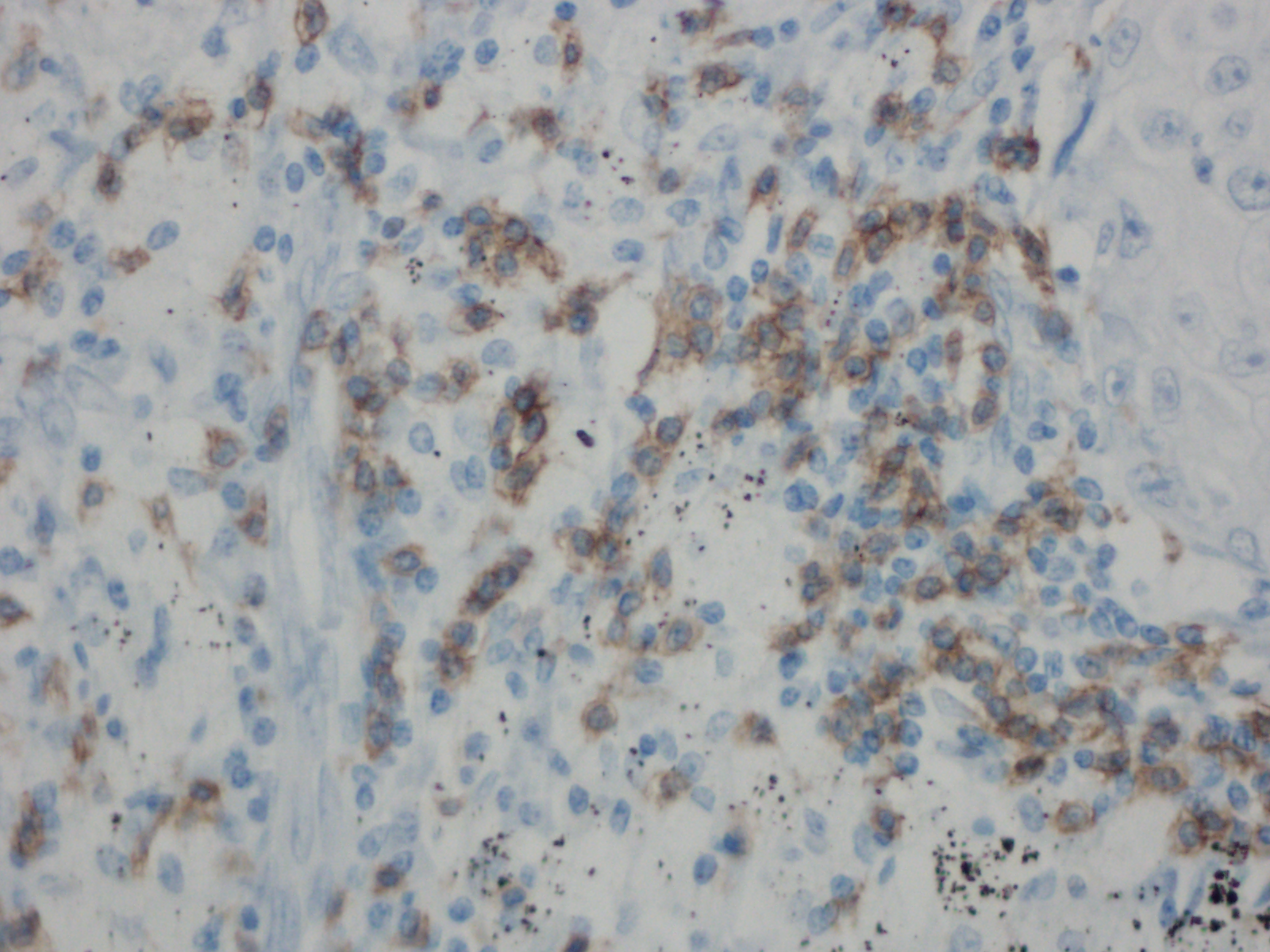

Supplement: Supplementary file 2 [file Data_Sheet_2.zip › primary tumorCD8.tif]

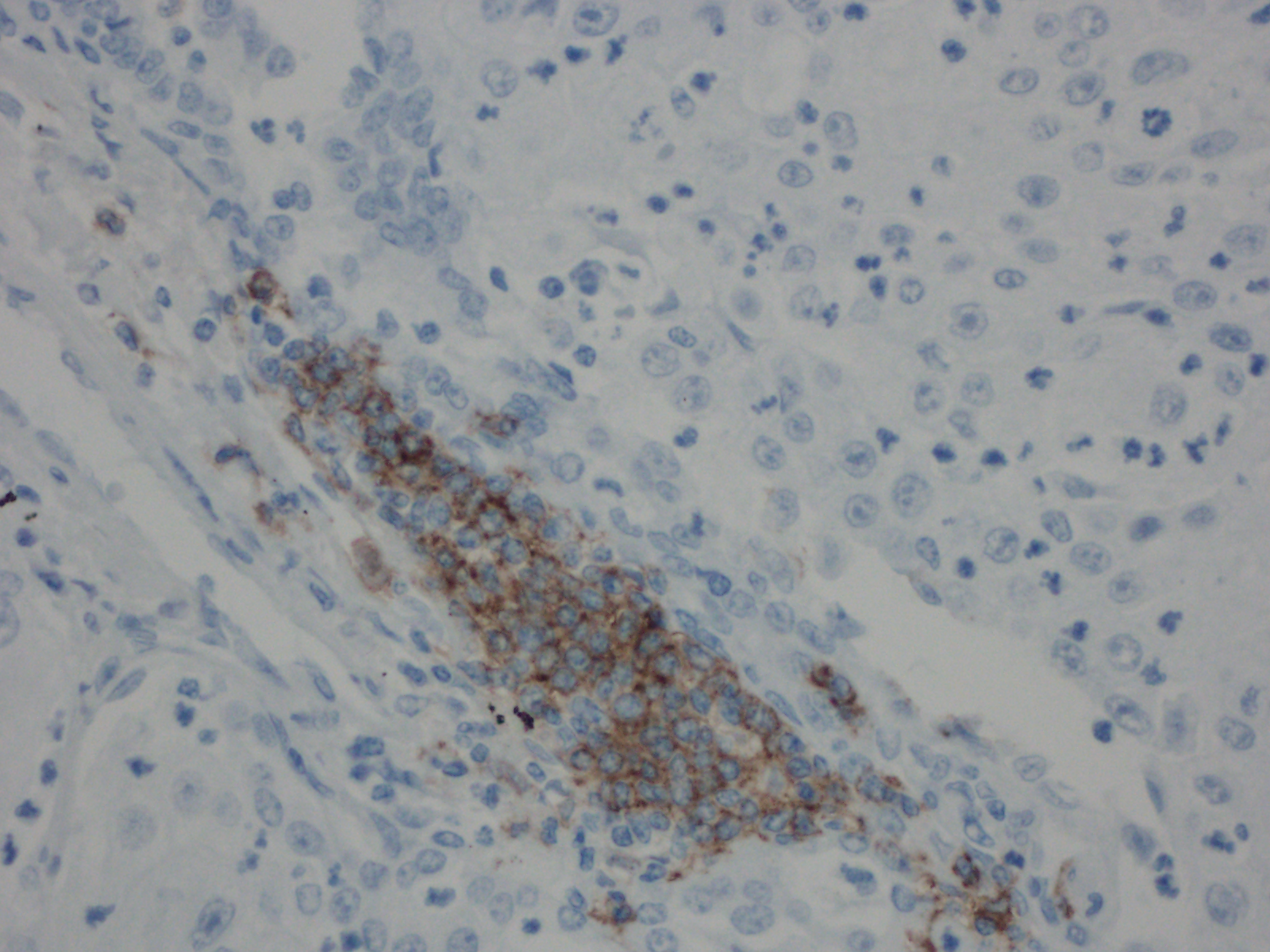

Supplement: Supplementary file 2 [file Data_Sheet_2.zip › primary tumorCD20.tif]

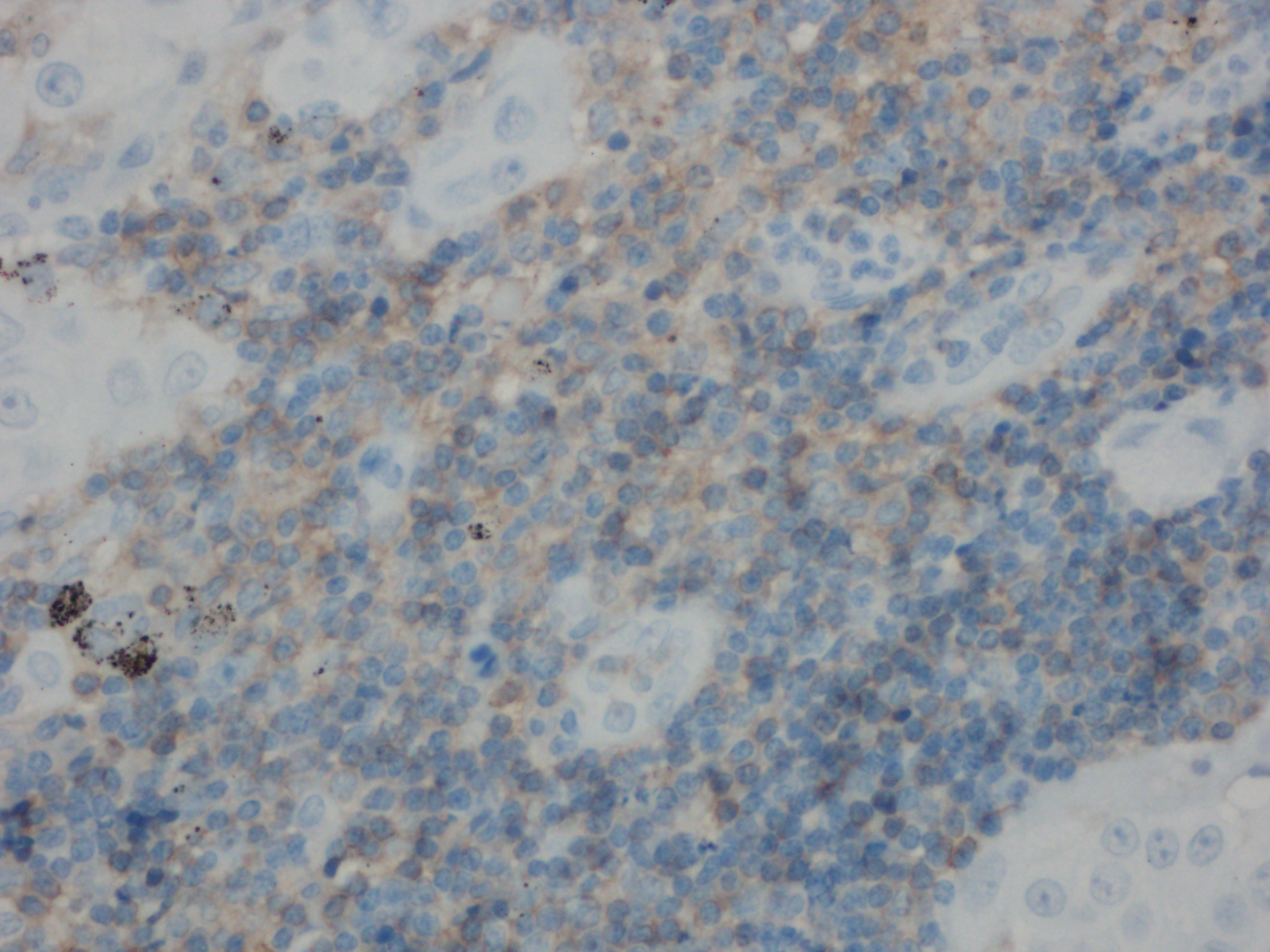

Supplement: Supplementary file 2 [file Data_Sheet_2.zip › the 4th LN CD4.tif]

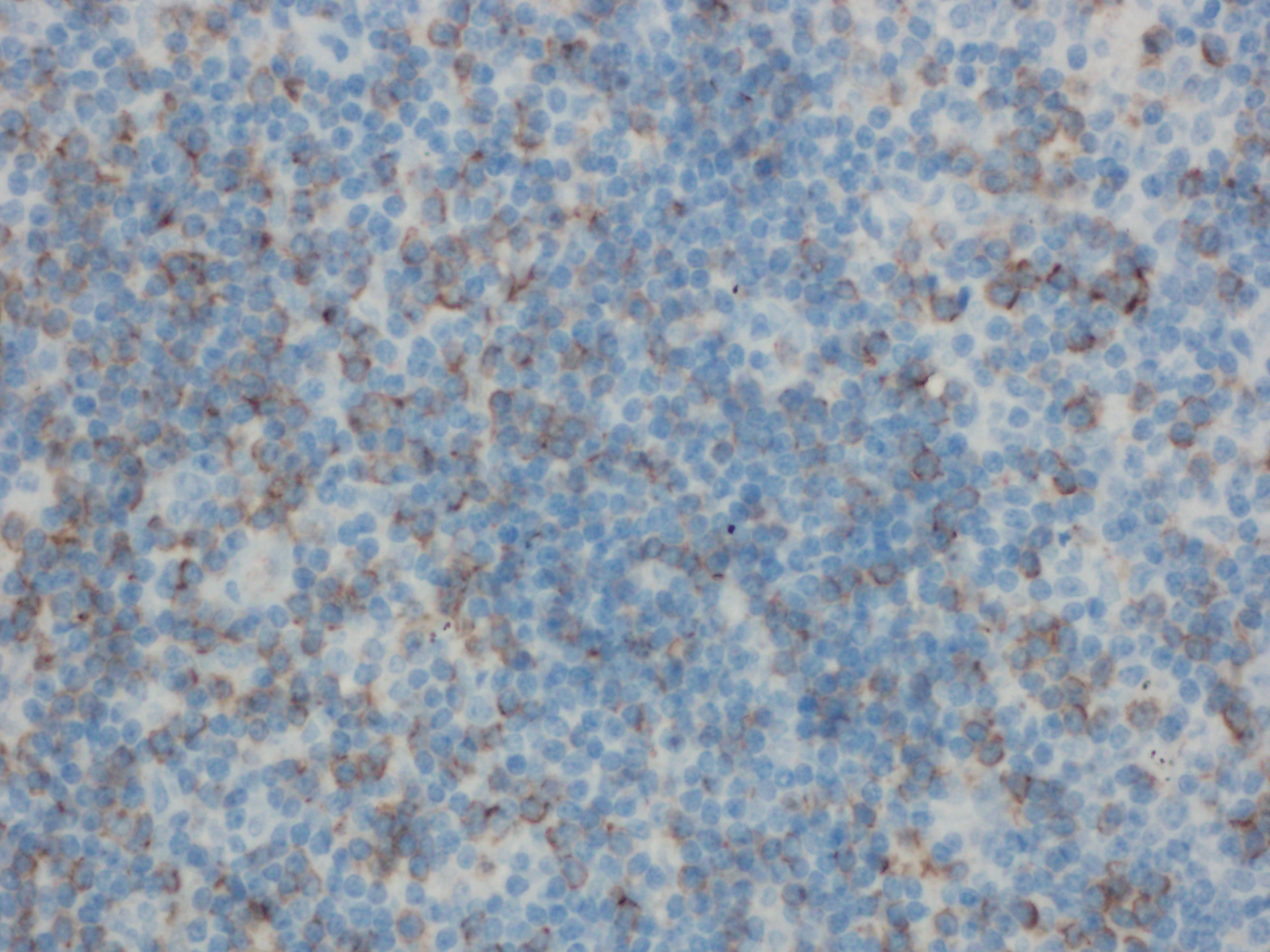

Supplement: Supplementary file 2 [file Data_Sheet_2.zip › the 4th LN CD8.tif]

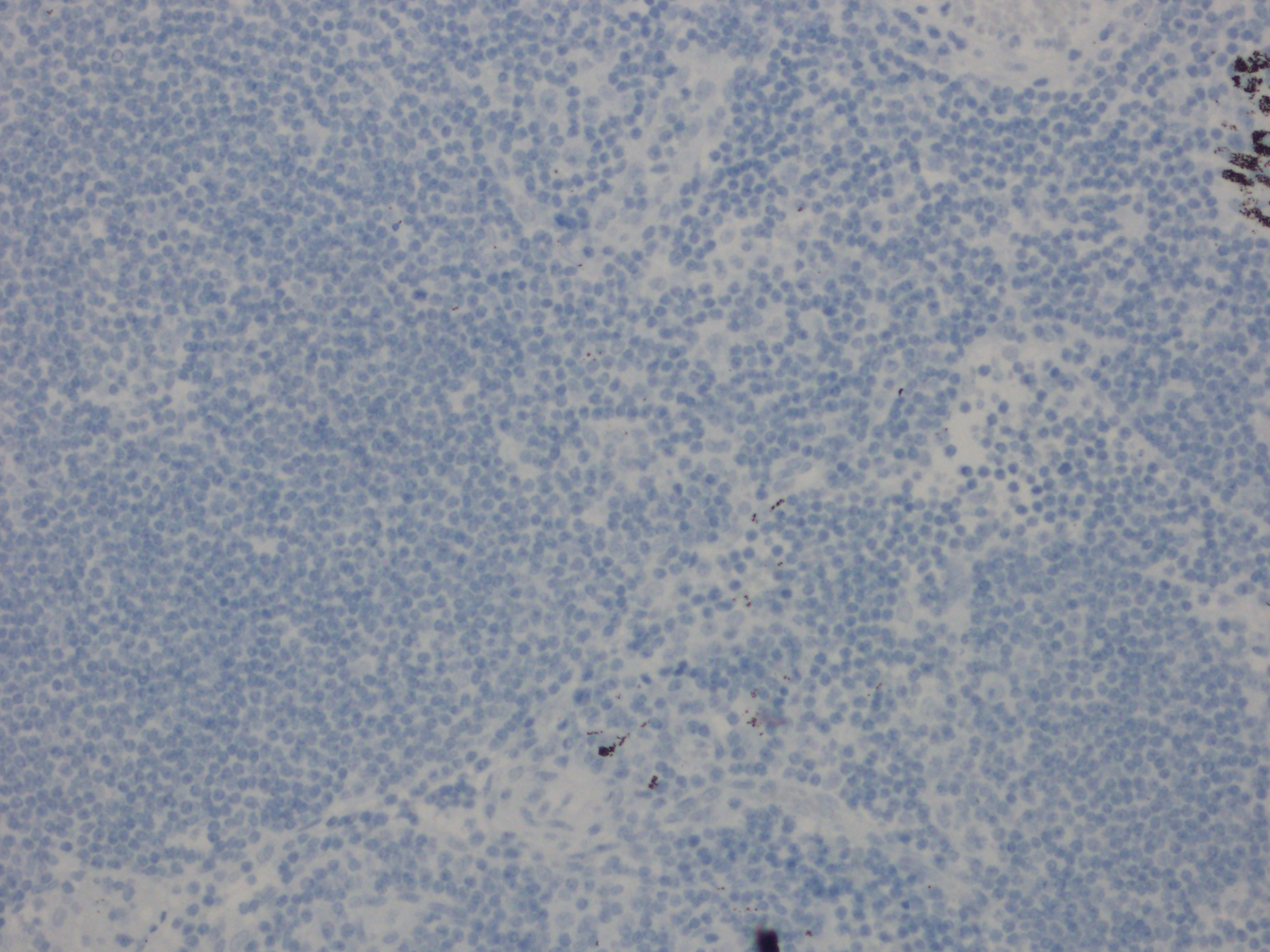

Supplement: Supplementary file 2 [file Data_Sheet_2.zip › the 4th LN CD20.tif]

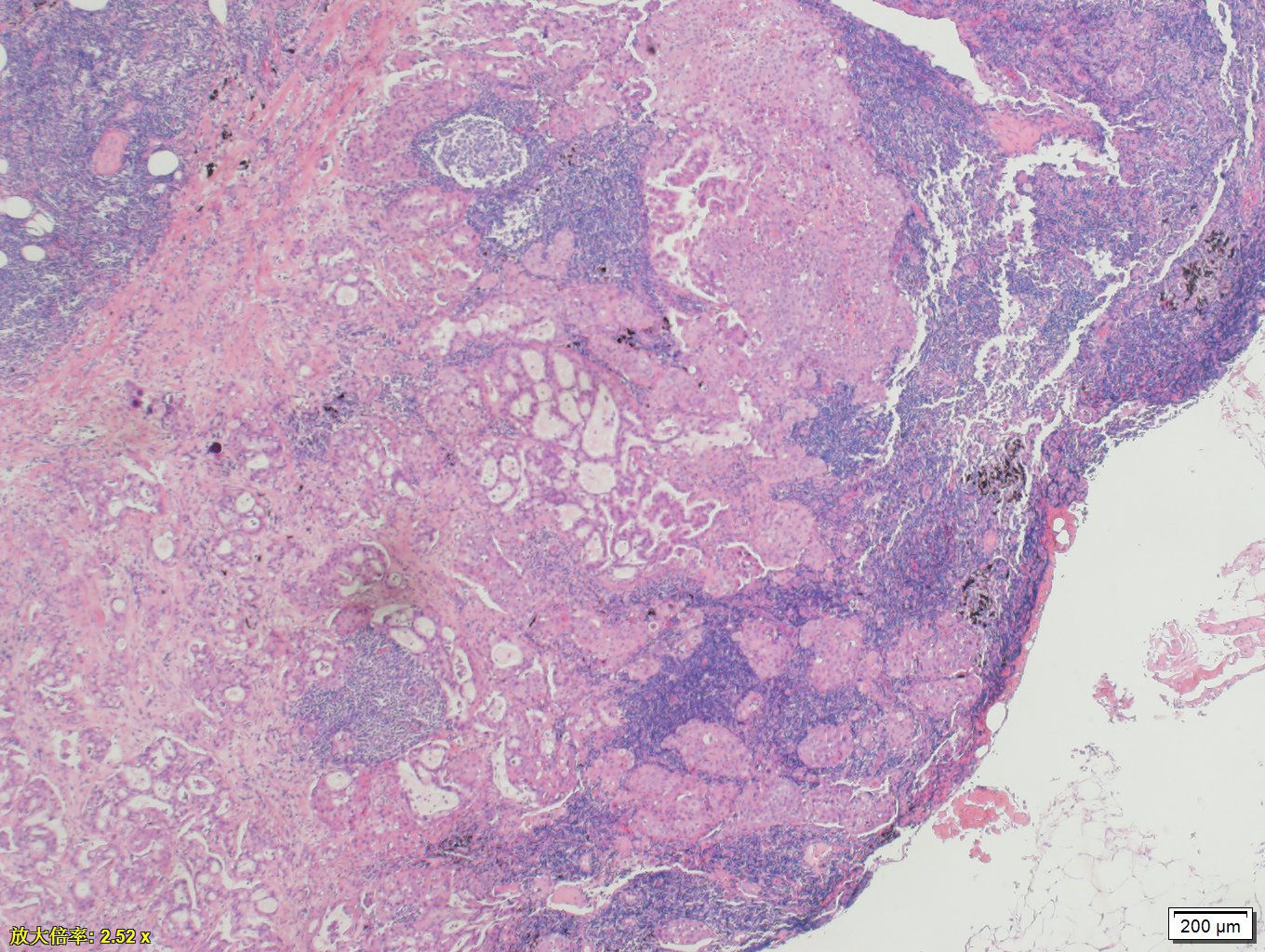

Supplement: Supplementary file 2 [file Data_Sheet_2.zip › the 4th LN.jpg]

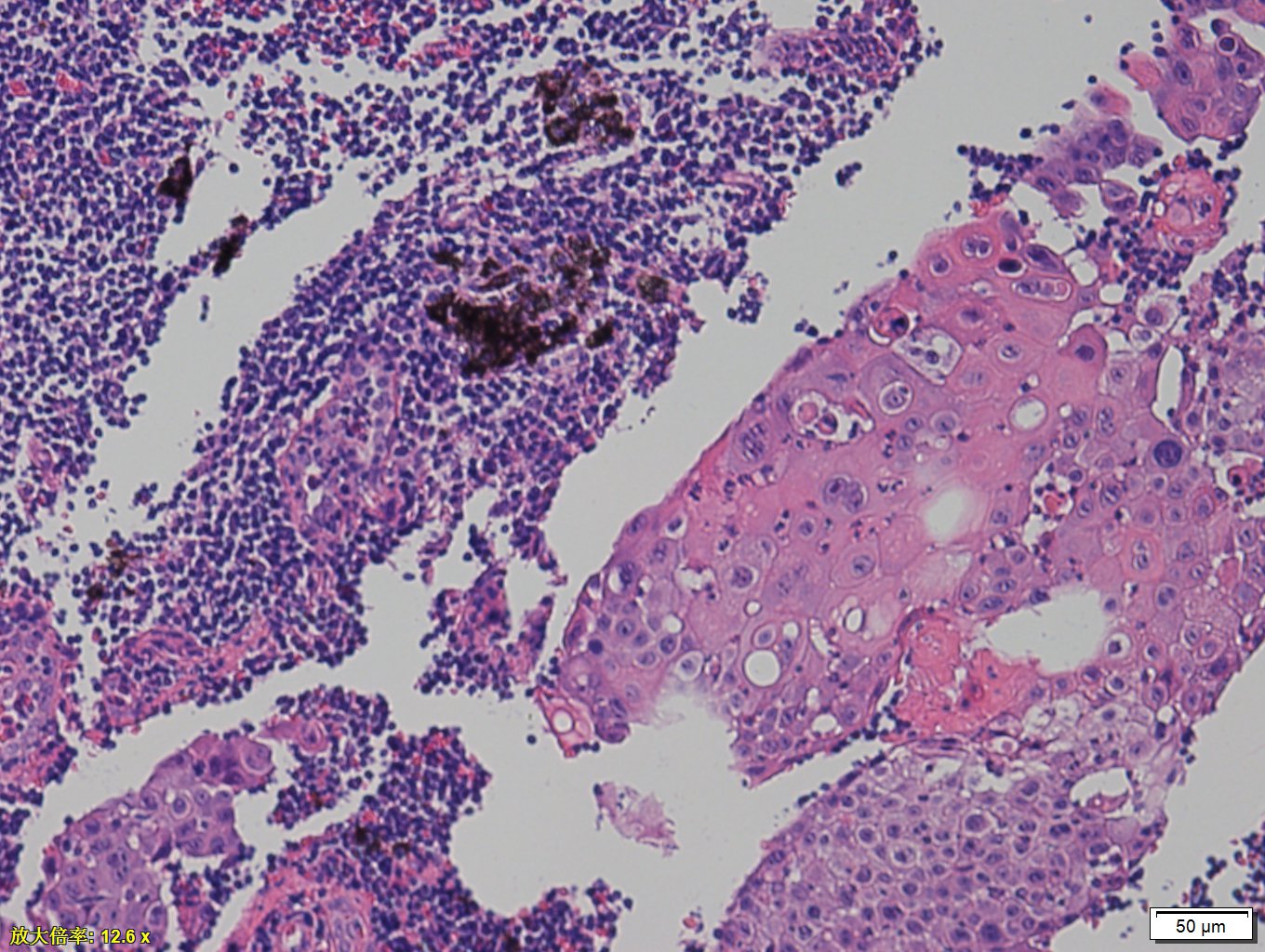

Supplement: Supplementary file 2 [file Data_Sheet_2.zip › the 10th LN.jpg]
